# Automatic Segmentation of the Nasolacrimal Canal: Application of the nnU-Net v2 Model in CBCT Imaging

**DOI:** 10.3390/jcm14030778

**Published:** 2025-01-25

**Authors:** Emre Haylaz, Ismail Gumussoy, Suayip Burak Duman, Fahrettin Kalabalik, Muhammet Can Eren, Mustafa Sami Demirsoy, Ozer Celik, Ibrahim Sevki Bayrakdar

**Affiliations:** 1 Department of Oral and Maxillofacial Radiology, Faculty of Dentistry, Sakarya University, Sakarya 54050, Turkey; 2 Department of Oral and Maxillofacial Radiology, Faculty of Dentistry, Inonu University, Malatya 44000, Turkey; 3 Independent Researcher, Sakarya 54100, Turkey; 4 Mathematics and Computer Science, Faculty of Science, Eskişehir Osmangazi University, Eskişehir 26040, Turkey; 5 Department of Oral and Maxillofacial Radiology, Faculty of Dentistry, Eskişehir Osmangazi University, Eskişehir 26040, Turkey

**Keywords:** artificial intelligence, cone beam-computed tomography, machine learning, neural networks, nasolacrimal duct

## Abstract

**Background/Objectives:** There are various challenges in the segmentation of anatomical structures with artificial intelligence due to the different structural features of the relevant region/tissue. The aim of this study was to detect the nasolacrimal canal (NLC) using the nnU-Net v2 convolutional neural network (CNN) model in cone beam-computed tomography (CBCT) images and to evaluate the successful performance of the model in automatic segmentation. **Methods:** CBCT images of 100 patients were randomly selected from the data archive. The raw data were transferred to the 3D Slicer imaging software in DICOM format (Version 4.10.2; MIT, Massachusetts, USA). NLC was labeled using the polygonal type of manual method. The dataset was split into training, validation and test sets in a ratio of 8:1:1. nnU-Net v2 architecture was applied to the training and test datasets to predict and generate appropriate algorithm weight factors. The confusion matrix was used to check the accuracy and performance of the model. As a result of the test, the Dice Coefficient (DC), Intersection over Union (IoU), F1-Score and 95% Hausdorff distance (95% HD) metrics were calculated. **Results:** By testing the model, DC, IoU, F1-Scores and 95% HD metric values were found to be 0.8465, 0.7341, 0.8480 and 0.9460, respectively. According to the data obtained, the receiver-operating characteristic (ROC) curve was drawn and the AUC value under the curve was determined to be 0.96. **Conclusions:** These results showed that the proposed nnU-Net v2 model achieves NLC segmentation on CBCT images with high precision and accuracy. The automated segmentation of NLC may assist clinicians in determining the surgical technique to be used to remove lesions, especially those affecting the anterior wall of the maxillary sinus.

## 1. Introduction

The nasolacrimal canal (NLC), part of the lacrimal drainage system, consists of a bony and a membranous canal. The bony part is flanked laterally by the sulcus lacrimalis and medially by the processus lacrimalis. It extends from the lower end of the lacrimal sac to the meatus nasi inferior, forming an angle of approximately 15 to 30 degrees with the coronal plane. The average width of the intraosseous canal is 4 mm, and its length ranges between 13 and 28 mm. The mucosa lining the lacrimal drainage system forms folds and sinuses, with the most crucial being the Hasner valve at the meatus nasi inferior [1,2,3].

Epiphora is a condition characterized by the overflow of tears due to partial or complete blockage of the NLC, which may be congenital or acquired [3,4,5]. Primary acquired dacryostenosis, an idiopathic fibroinflammatory obstruction, is the leading cause. Secondary acquired dacryostenosis often results from infections, trauma, surgery, tumors or radiation therapy [4,6,7].

Over the years, various imaging methods have been utilized to examine the morphological changes in the NLC, as well as to detect, treat and follow up duct and sac diseases. These methods include conventional dacryocystography (DCG) [8], digital subtraction dacryocystography (DS-DCG) [9], computed tomography (CT) [4], computerized tomography–dacryocystography (CT–DCG) [10], magnetic resonance imaging (MRI) [11], and cone beam-computed tomography (CBCT) [12]. Accurate detection of the NLC is essential for maxillofacial surgical procedures, as damage to the lower opening of the NLC or the anterior wall of the lacrimal sac may occur during surgery. Therefore, selecting an imaging method that is both accurate and effective is crucial [13,14].

The widespread use of three-dimensional (3D) imaging methods, particularly cone beam-computed tomography (CBCT) in dentistry, has addressed the limitations of two-dimensional (2D) imaging [15]. In addition, the effectiveness and success of artificial intelligence algorithms have been enhanced by the use of CBCT and other 3D imaging methods. Diagnostic images have become a primary resource for developing AI systems, which can be used to detect pathological changes, segment craniofacial anatomical structures, and classify maxillofacial tumors and cysts [16,17,18,19,20]. For example, Widiasri et al. [21] developed an AI model for detecting alveolar bone thickness and the mandibular canal to aid in dental implant planning. In another study, AI algorithms were utilized to assess growth and development based on cervical vertebra stages [22]. Zhang et al. [23] successfully predicted postoperative facial swelling in orthognathic surgery patients using a convolutional neural network (CNN)-based model. These capabilities are invaluable for clinicians during both intraoperative and postoperative decision-making. AI technology has the potential to minimize errors, assist in surgical treatment planning, reduce diagnosis and treatment times and improve overall operational efficiency [24,25,26].

However, most current deep learning algorithms require manual intervention for lesion detection and segmentation, making the process time-consuming and less practical for routine clinical use [27,28]. To address these limitations and improve usability, researchers are focusing on developing algorithms that can fully automate these steps [16,29,30].

To the best of our knowledge, no previous study has evaluated the automatic segmentation of NLC on CBCT images using the nnU-Net v2. This model simplifies and streamlines the system by employing a systematic approach, avoiding the need for additional network structures and eliminating system complexity. Furthermore, the nnU-Net v2 model can be adapted into a semi-automatic system by incorporating manual inputs, enabling external intervention to address deficiencies and enhance network performance [31].

The aim of this study is to develop a CNN algorithm utilizing the nnU-Net v2 architecture and evaluate its performance in automatically detecting the NLC in axial CBCT images. A successful model could serve as a reliable tool for clinicians, aiding in the assessment of the relationship between pathologies affecting the paranasal sinuses, nasal region and the NLC. This could improve preoperative planning and intraoperative guidance, ultimately enhancing surgical outcomes.

Additionally, the development of an automatic segmentation tool for the NLC could streamline the diagnostic process, reducing the need for manual segmentation and saving time for radiologists and surgeons. This could lead to increased efficiency in clinical workflows. Our hypothesis is that a CNN algorithm utilizing the nnU-Net v2 architecture will achieve high accuracy and efficiency in automatically detecting the NLC in axial CBCT images.

## 2. Materials and Methods

### 2.1. Study Design

In this study, the CBCT data of patients who applied to Inonu University Faculty of Dentistry, Oral and Maxillofacial Radiology Clinic for any reason were used. Since the aim is to develop and validate an AI for automatic NLC detection, this study consists of three phases: development, training and testing. CBCT scans used full convolutional neural network algorithms (nnU-Net v2) for 3D automated detection of NLC. The AI Checklist in Medical Imaging (CLAIM) and Standards for Reporting of Diagnostic Accuracy Studies (STARD) were used in the preparation of this article. The study was retrospectively designed and all procedures were carried out in accordance with the Declaration of Helsinki and similar ethical standards. The Inonu University Non-Interventional Clinical Research Ethics Committee approved the study protocol (decision no: 2024/6575). As a routine protocol, all patients in the CBCT archive had been informed and provided written consent regarding the use of their data for scientific research.

### 2.2. Data

This study’s sample size was determined using the G*Power software (version 3.1.9.7; Franz Faul, University of Kiel, Kiel, Germany). With an α error probability of 0.05 and a power of the study of 0.95, the actual power of the study was calculated to be 95% when at least 100 samples were included [32]. The final sample was obtained from 100 CBCT images (200 right and left NLC) of 50 males and 50 females randomly selected using archival records. The ages of the patients ranged from 18 to 72 years, and the mean age was 52 years. The inclusion criteria are as follows:

- Individuals over 18 years of age.
- Individuals without any syndrome or bone disease.
- Clearly identified images of NLC’s bone boundaries.

The exclusion criteria are as follows:

- Individuals with known pre-existing infection, neoplasm and malformations associated with NLC.
- Individuals who have undergone surgical operations and trauma involving the maxillofacial region and NLC.
- Images with motion or metal artifacts that prevent NLC from being displayed and degrade diagnostic quality.

### 2.3. Obtaining and Evaluating CBCT Images

The scans were performed using a NewTom 5G CBCT machine (Quantitative Radiology, Verona, Italy); 110 kVp, 1–11 mA, 3.6 s, 8 × 8 cm^2^, 12 × 8 cm^2^ and 15 × 12 cm^2^ field of view (FOV) were obtained with 0.2 mm^3^–0.3 mm^3^ voxel size parameters. CBCT images were taken between 2021 and 2023 for various reasons.

### 2.4. Ground Truth

After reconstruction of the raw data, DICOM images were transferred to the 3D Slicer imaging software (Version 4.10.2; MIT, Cambridge, MA, USA) for manual segmentation. NLC was manually labeled in the coronal, sagial and axial planes using 3D Slicer imaging software, an open source program. The labeled DICOM data were converted to NIfTI (Neuro Imaging Information Technology Initiative) format and exported for processing. The ground truth was defined by the consensus of two maxillofacial radiologists (E.H. and I.G.), both of whom had at least 6 years of experience in maxillofacial radiology. Upon completion of the manual segmentation process, it was checked by senior individuals (I.S.B. and F.K.) who had at least 10 years of experience in maxillofacial radiology and reached full agreement on all labels.

### 2.5. Testing Data

**Figure 1:**
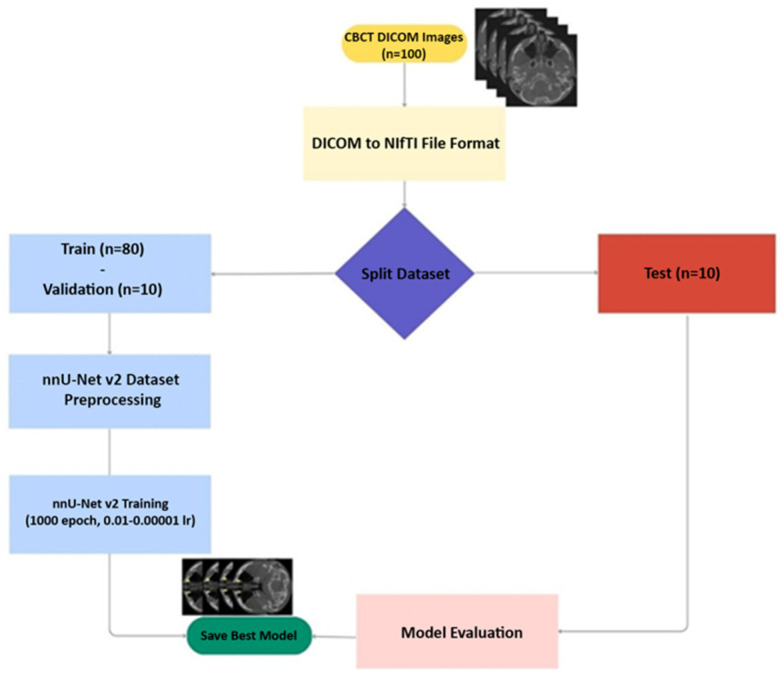
Workflow model of automatic segmentation of the nasolacrimal canal.

After screening the best-performing model through comprehensive comparison, 100 CBCT datasets were divided into training, validation and test sets according to the ratio of 8:1:1 (Figure 1).

### 2.6. Model

The optimal model was used for 10-fold cross-validation on the training set, and the validation and test sets were evaluated. Training model and parameters: nnU-Net v2 based FCNN model, 1000 epochs, 0.00001 learning rate. The algorithm of the nnU-Net v2 model for automatic segmentation of NLC was developed in a Python environment (v3.6.1; Python Software Foundation, Wilmington, DE, USA) using the PyTorch library. The CranioCatch AI software (Version 2.1; CranioCatch, Eskisehir, Turkey) was used in the deep learning model development and training process described by Bayrakdar et al. [32].

U-Net was introduced to the world in 2015 by Olaf Ronneberber, Philip Fischer and Thomas Brox in an article titled “U-Net: Convolutional Networks for Biomedical Image Segmentation” for better segmentation, particularly in biomedical images [33]. The FCNN (Fourier Convolution Neural Network) model used in this study, nnU-Net (Neural Networks U-Net) v2, is an improved version of the U-Net architecture. Large datasets are needed for training in classical convolutional neural network models. The images in these datasets are labeled and presented to the network, and the network recognizes the images with this label information. This labeling process is particularly challenging for biomedical images due to their pixel-based nature, requiring significant human and hardware resources. Unlike classical CNN models, U-Net offers a unique architecture for pixel-based image segmentation, addressing these challenges.

### 2.7. Evaluation

The model’s performance in the automated 3D segmentation of the NLC on CBCT volumes was evaluated using a confusion matrix. The confusion matrix is an essential performance evaluation tool for measuring algorithm accuracy and establishing success parameters. Probabilities of segmentation models were derived from the true positive (TP), true negative (TN), false positive (FP) and false negative (FN) values in the confusion matrix. The test results yielded the confusion matrix for the nnU-Net v2 model.

Recall (sensitivity) and precision metrics of the proposed model are calculated with the expressions in the confusion matrix (Table 1). In this present study, the results are presented with Dice Coefficient (DC), F1-Score and Intersection over Union Intersection (IoU) metrics. Additionally, the area under curve (AUC) value and the 95% Hausdorff distance (95% HD) were calculated in mm. These metrics are frequently used in the literature to measure the success of segmentation, and information about the metrics is given in this section.

## 3. Results

**Figure 2:**
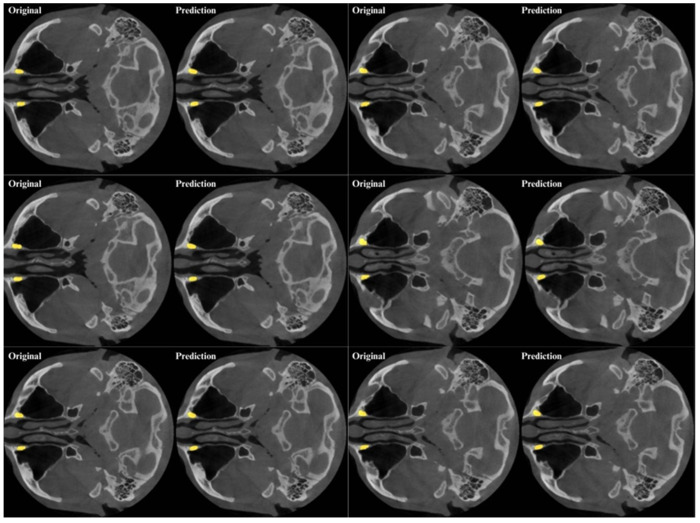
Automatic segmentation of nasolacrimal canal using artificial intelligence model in axial CBCT slices.

Within the scope of this study, the nnU-Net v2 model, which is one of the deep learning networks, was used for NLC segmentation. A total of 80% of the dataset was determined as the training group, 10% as the validation group, and 10% as the test group. The training was completed in an average of 1000 epochs. In all models, “Adam” was used as the optimization algorithm. ReLU activation is used in the proposed model. The training parameters of the nnU-Net v2 model are given in Table 2. The predictive analysis of the proposed nnU-Net v2 model is shown in Figure 2.

**Figure 3:**
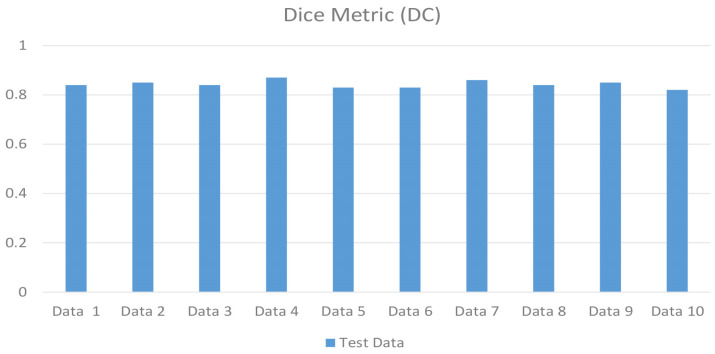
Dice confidence (DC) scores of the test data.

**Figure 4:**
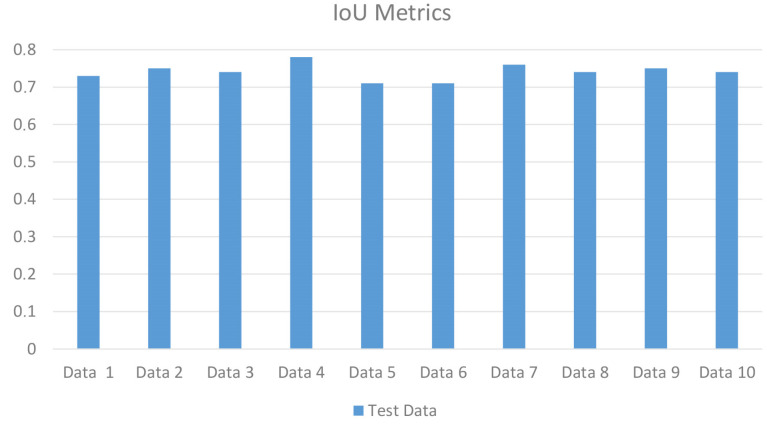
IoU metrics of the test data.

The values of the precision and recall metrics were calculated as 0.7888 and 0.9168, respectively (Table 1). According to the results, the highest DC, IoU, F1-Score and 95% Hausdorff distance values after the segmentation performed in the nnU-Net v2 architecture were found to be 0.8465, 0.7341, 0.8480 and 0.9460, respectively (Table 1). The DC and IoU metrics of the test data are shown in Figure 3 and Figure 4.

**Figure 5:**
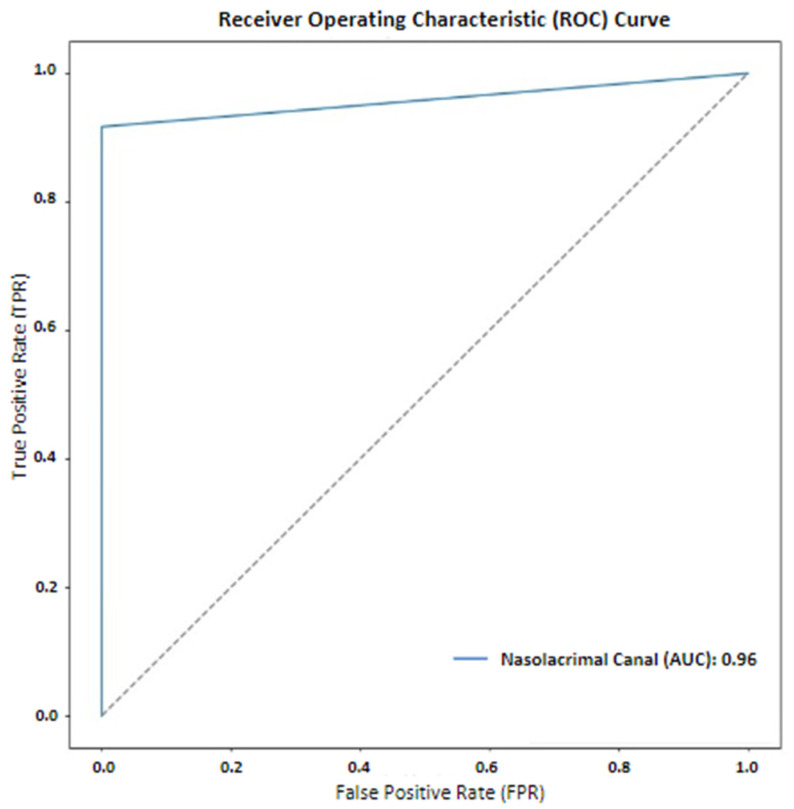
Receiver-operating characteristic (ROC) curve and AUC value.

**Figure 6:**
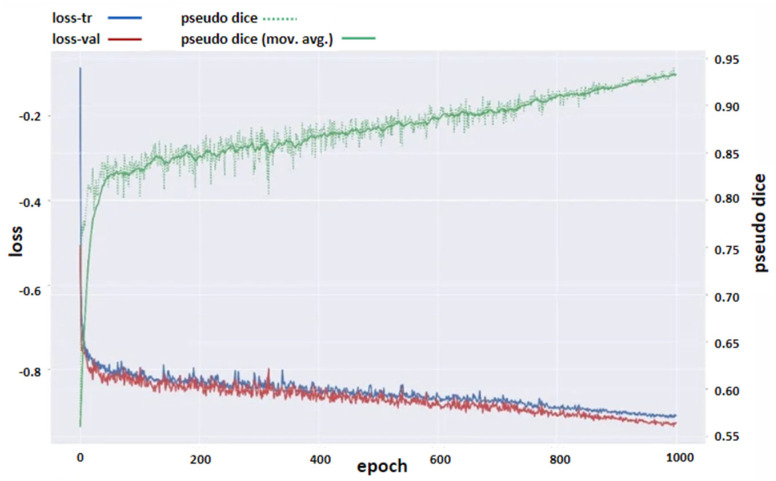
Dice score and loss function values at each number of cycles of the model.

According to the test results, the receiver-operating characteristic (ROC) curve was drawn and the AUC value under the curve was determined to be 0.96 (Figure 5). In order to have more information about the training of the model, a graph of the dice score and loss function values at each number of cycles was created, starting from the first number of cycles of the model (Figure 6).

## 4. Discussion

Paranasal sinuses and nasal anatomical structures are closely related to the NLC. Due to this anatomical proximity, paranasal sinus diseases and pathologies involving the nasal region often affect the NLC [34]. In cases where the anterior wall and floor of the maxillary sinus are involved, surgical intervention can become challenging. In such situations, the anterior wall of the maxillary sinus can be accessed using the prelacrimal approach [35]. This technique may lead to the removal of the NLC’s bony component and displacement of the mucosa. To determine whether NLC dislocation or resection is required, the distance between the anterior wall of the maxillary sinus and the NLC should be considered [36]. Automatic segmentation of the NLC can help clinicians practically and easily assess the distance between the NLC and the anterior wall of the maxillary sinus, aiding in determining the appropriate surgical technique for planned interventions in the maxillary sinus and nasal region. Moreover, accurate segmentation of the NLC provides crucial guidance for maxillofacial surgical procedures and pathology evaluations, serving as an essential tool for preoperative and intraoperative planning and ultimately enhancing surgical outcomes. This study introduces a new automatic segmentation tool that uses the nnU-Net v2-based AI model to achieve high accuracy and efficiency in NLC segmentation. It is innovative in that it is one of the first studies in the literature to use AI for NLC segmentation.

To the best of our knowledge, there are no studies on NLC segmentation using AI in the literature yet. This study aimed to evaluate the performance of the nnU-Net v2 model in segmenting the NLC from CBCT scans. The DC was 0.8465, indicating successful NLC segmentation. An IoU metric of 0.7341 further confirmed accurate segmentation, as an IoU above 0.5 is typically deemed successful [37]. The F1-Score of 0.8480 demonstrated the model’s high sensitivity and precision. The ROC curve indicated an AUC value of 0.96, highlighting the model’s strong ability to accurately distinguish the NLC from CBCT images. These findings confirmed our hypothesis (H1). This suggests the model’s potential for use in diagnosing NLC, planning treatment and evaluating NLC-related diseases before maxillofacial surgery.

Manual labeling of CBCT scans is labor-intensive, time-consuming and costly. Even with semi-automatic labeling, operator-dependent errors are unavoidable. Precise segmentation can aid forensic science by providing detailed anatomical information that is critical for identifying individuals and understanding trauma mechanisms. In addition, precise automatic segmentation can process large datasets in a fraction of the time, significantly reducing the workload for researchers and clinicians. Moreover, automatic segmentation provides consistent and reproducible results. This consistency is crucial for comparative studies and longitudinal research where precise measurements are essential. Thus, there is a growing need for the fully automated labeling of CBCT scans [38,39]. Preda et al. [40] investigated automatic segmentation of the maxillofacial complex using CBCT data. They reported that automated segmentation takes less than a minute and is 204 times faster than manual segmentation. In their study, they reported the mean values of DSC, 95% HD and IoU metrics as 0.926, 0.621 and 0.862, respectively. The findings showed high similarity between automatically and manually segmented CBCT data. These results highlight that automated segmentation increases efficiency in the workflow and saves time [41].

AI and DL algorithms are used for different purposes in medical imaging. These include image segmentation and preprocessing, disease detection and diagnosis, personalized treatment planning, predictive analytics, quality control, monitoring and follow-up [42]. In particular, DL models can be used to follow the radiotherapy process by automating the segmentation of organs and tumors. Moreover, DL algorithms enable adaptive radiotherapy, where treatment plans can be continuously optimized over time according to changes in a patient’s anatomy. This adaptive approach ensures that radiation is delivered with the highest possible accuracy, improving treatment outcomes while minimizing side effects [43]. Different deep learning models, the nnU-Net model and its variants are widely used in the literature for similar studies (Table 3). This model has been employed for segmenting various anatomical structures and detecting pathologies, such as mandibular canal abnormalities [34], maxillary sinus pathologies [41] and the classification of jaw lesions [44], have been carried out with this model.

Zhu et al. [49] utilized the nnU-Net model for diagnosing impacted teeth, dental caries, crowns, missing teeth and residual roots. However, there are no studies yet on the automatic segmentation of NLC. Three-dimensional imaging of the NLC not only enhances anatomical understanding but also facilitates the creation of virtual models to guide surgical planning in the maxillofacial region. In this context, the precise and comprehensive segmentation of the NLC is a crucial preliminary step.

Shi et al. [50] employed the nnU-Net model to segment CBCT data from 48 patients with class II malocclusion who underwent orthognathic surgery. They evaluated the success of automatic segmentation in identifying condylar changes. The DC values for the automatic segmentation of the maxilla, mandible and condylar changes were 0.9263, 0.9387 and 0.971, respectively. This study concluded that artificial intelligence exhibits high sensitivity in detecting condylar changes [50]. Oztürk et al. [37] performed maxillary sinus segmentation on CBCT images with the U-Net network. They used F1-Score and IoU metrics to evaluate the model’s performance. The segmentation results demonstrated an F1-Score of 0.9784 and an IoU value of 0.9275. These metric values confirmed the model’s success in maxillary sinus segmentation [37]. As highlighted in the aforementioned studies, artificial intelligence-based segmentation serves diverse purposes, and its performance can be evaluated using various metrics.

Morita et al. [17] assessed the segmentation of eight anatomical parameters in the facial region using a 2D U-Net model on CT data. The mean DC values for maxilla, mandible, right zygoma segmentation and left zygoma segmentation were found to be 0.909 ± 0.036, 0.984 ± 0.017, 0.936 ± 0.029 and 0.926 ± 0.049, respectively. The model showed high success. However, the average DC values for the nasal bone (0.838 ± 0.084) and frontal bone (0.858 ± 0.060) showed lower accuracy compared to the others. They attributed this to the fact that the nasal bone is thinner and smaller than other bones. They also reported that sutures on the nasal and frontal bones would affect segmentation success [17]. In this present study, the DC value for NLC segmentation was comparable to the values reported by Morita et al. [17] for the nasal and frontal bones, yet it exhibited lower success rates compared to other anatomical structures.

Yağmur et al. [45] utilized the nnU-Net v2 model, similar to the methodology employed in this study. The DC value for mandibular canal segmentation in their study was reported as 0.76. This current study demonstrated that NLC segmentation achieved higher success using the same model. However, success performance was found to be lower in this current study compared to other studies [40,41,51,52]. This discrepancy can be attributed to the anatomical connection of the NLC’s bony component with adjacent spaces. Especially, its connection to the lower meatus is wide and angulated, making manual segmentation difficult. Consequently, accurate segmentation proves challenging in cases where distinct bony boundaries are not clearly discernible.

Chang et al. [48] developed an automatic method for staging periodontitis using a deep learning hybrid framework. They proposed a novel approach that combines deep learning architecture for detection with conventional CAD (Computer-Aided Diagnosis) processing for classification. The deep learning model was employed to detect the radiographic bone level as a simple structure for the entire jaw on panoramic radiographs. They found that this hybrid framework, which integrates deep learning and traditional CAD methods, demonstrated high accuracy and excellent reliability in automatically diagnosing periodontal bone loss and staging periodontitis.

As evident from previous studies, the U-Net network architecture has been employed for various purposes in medicine and dentistry [17,37,45,46]. High achievement performance with correct training and appropriate data is one of the reasons why this model is preferred. U-Net network architecture provides success even with very limited data and has superior performance speed compared to other models. Due to these qualified features, it is frequently preferred in the biomedical field, especially for segmentation purposes [46,52].

This study had several limitations. Firstly, while the developed model allowed for the bone canal border of the NLC to be clearly determined, it did not achieve the same success rate in distinguishing the membranous canal. The use of multimodal segmentation models (CT-MR, CBCT-MR) to improve soft tissue contrast may be considered in the future, which can enhance the model’s robustness and performance. Furthermore, issues related to low resolution could be addressed. Secondly, CBCT data were obtained from a single institution. Data from different centers would increase heterogeneity and allow for the results to be generalized. Finally, there is the difficulty of separating the NLC from the spaces it connects. Therefore, using more advanced multi-stream (multi-angle, multi-scale and multi-modality) models would be beneficial for improving the accuracy of NLC segmentation in 3D images.

## 5. Conclusions

This study evaluated the nnU-Net v2 model for segmenting the NLC in CBCT images using a dataset of 100 patients. The DC value of 0.8465 and the AUC value of 0.96 demonstrated high accuracy. The complex anatomical structure of the maxillofacial region makes the manual labeling of CBCT images laborious and time-consuming in practice. The NLC is an important anatomical structure that must be considered before surgical procedures in this region, particularly due to its proximity to the anterior wall of the maxillary sinus. The proposed nnU-Net v2 model demonstrated promising performance in segmenting the NLC on CBCT images. Our results suggest that the automated segmentation of the nasolacrimal canal can facilitate clinical decision-making, improve work efficiency and save time during diagnosis and treatment.

Future efforts will focus on addressing the current limitations by expanding the dataset with additional CBCT images and labels. Furthermore, specific adjustments to the nnU-Net v2 model, such as hyperparameter tuning, enhanced data augmentation techniques and architectural modifications (e.g., attention mechanisms), are planned to further improve the DC and other performance metrics. These refinements aim to develop a more robust and accurate model for clinical applications.

## Figures and Tables

**Table 1:** Metrics used to evaluate the performance of the nnU-Net v2 model, and the results obtained.

Metrics	Metric Formula	Metric Value
True Positive		16,297.7
False Positive		4214.2
False Negative		1624.5
Precision	TP/(TP + FP)	0.7888
Recall (Sensitivity)	TP/(TP + FN)	0.9168
Dice Coefficients (DC)	(2 × T P)/(2 × T P + F P + F N)	0.8465
Intersection over Union (IoU)	(|A∩B|)/(|A∪B|)	0.7341
F1-Score	2 × (Precision × Recall)/(Precision + Recall)	0.8480
95% Hausdorff Distance (95%HD) mm	*d*_H95_(A, B) = *max*(*d*_95_(A, B), *d*_95_(A, B))	0.9460

Notes: TP: True Positive, FP: False Positive, FN: False Negative.

**Table 2:** Training parameters and value of the proposed nnU-Net v2 model.

Parameter	Value
Model	NnU-Net v2
Epoch	1000
Batch Size	2
Learning Rate	0.00001
Optimization	ADAM
Activation	ReLU

**Table 3:** A summary of several studies in the literature related to automatic segmentation of dental structures.

Authors	Aim	Sample	Segmentation Model	Imaging Method	Evaluation Metrics
Ozturk [37]	The aim of this study is to develop a deep learning-based method to perform maxillary sinus segmentation using CBCT images.	100 Scans	U-Net	CBCT	F-1 Score: 0.9784 IoU: 0.9275
Preda et al. [40]	This present study investigated the accuracy, consistency and time-efficiency of a novel deep convolutional neural network (CNN)-based model for the automated maxillofacial bone segmentation from CBCT images.	144 Patients	U-Net	CBCT	DC: 0.926 %95 HD: 0.621 IoU: 0.862
Shi et al. [44]	This study proposes an automated method to measure condylar changes in patients with skeletal class II malocclusion following surgical orthodontic treatment.	48 Patients	nnU-Net	CBCT	Maxilla DC: 0.9263 Mandible DC: 0.9387 Condyle DC: 0.971
Yağmur et al. [45]	The aim of this study is to evaluate the mandibular canal with CBCT using a deep learning approach.	300 Patients	nnU-Net v2	CBCT	DC: 0.76
Ascı et al. [46]	The purpose of this study was to evaluate the effectiveness of dental caries segmentation on the panoramic radiographs taken from children in primary dentition, mixed dentition and permanent dentition with AI models developed using the deep learning method.	6075 Patients	U-Net	Panoramic Radiographs	Sensitivity: 0.8269 Precision: 0.9123 F-1 Score: 0.8675
İçöz et al. [47]	The aim of this study was to evaluate the effectiveness of an AI system in the detection of roots with apical periodontitis on digital panoramic radiographs.	306 Scans	YOLOv3	Panoramic Radiographs	Sensitivity: 98% Specificity: 56% F-1 Score: 71%
Chang et al. [48]	The aim of this study was to develop an automated method for diagnosing periodontal bone loss for staging periodontitis on dental panoramic radiographs using the deep learning hybrid method for the first time.	340 Scans	Mask R-CNN	Panoramic Radiographs	Periodontal Bone Level IoU: 0.88 Accuracy: 0.92 DC: 0.93 Cementoenamel Junction Level IoU: 0.84 Accuracy: 0.87 DC: 0.91 Teeth and Implants IoU: 0.83 Accuracy: 0.87 DC: 0.91

## Data Availability

The original contributions presented in this study are included in the article. Further inquiries can be directed to the corresponding author.
